# Strategies for the Construction of Cassava Brown Streak Disease Viral Infectious Clones

**DOI:** 10.1007/s12033-018-0139-7

**Published:** 2018-11-27

**Authors:** C. R. A. Duff-Farrier, D. R. Mbanzibwa, S. Nanyiti, H. Bunawan, J. L. Pablo-Rodriguez, K. R. Tomlinson, A. M. James, T. Alicai, S. E. Seal, A. M. Bailey, G. D. Foster

**Affiliations:** 1 School of Biological Sciences, University of Bristol, 24 Tyndall Ave, Bristol, BS8 1TQ UK; 2 Mikocheni Agricultural Research Institute (MARI), P.O. Box 6226, Dar es Salaam, Tanzania; 3 Present Address: Institute of Systems Biology (INBIOSIS), Universiti Kebangsaan Malaysia, 43600 UKM Bangi, Selangor Darul Ehsan Malaysia; 4 Present Address: Department of Genetical Engineering, Centre for Research and Advanced Studies (CINVESTAV), Campus Irapuato, Km 9.6 libramiento Norte, Carretera Irapuato-León, Irapuato, 36824 Guanajuato, Mexico; 5 National Crops Resources Research Institute (NaCRRI), P.O. Box 7084, Kampala, Uganda; 6 Agriculture, Health and Environment Department, Natural Resources Institute, University of Greenwich, Chatham, Kent ME4 4TB UK

**Keywords:** Virus, Cassava, Cassava brown streak virus, Ugandan cassava brown streak virus, Infectious clones

## Abstract

Cassava brown streak disease (CBSD) has major impacts on yield and quality of the tuberous roots of cassava in Eastern and Central Arica. At least two *Potyviridae* species cause the disease: *Cassava brown streak virus* (CBSV) and *Ugandan cassava brown streak virus* (UCBSV). Cloned viral genome sequences known as infectious clones (ICs) have been important in the study of other viruses, both as a means of standardising infectious material and characterising viral gene function. IC construction is often technically challenging for *Potyviridae* due to sequence instability in *E. coli*. Here, we evaluate three methods for the construction of infectious clones for CBSD. Whilst a simple IC for in vitro transcription was made for UCBSV isolate ‘Kikombe’, such an approach failed to deliver full-length clones for CBSV isolates ‘Nampula’ or ‘Tanza’, necessitating more complex approaches for their construction. The ICs successfully generated symptomatic infection in the model host *N. benthamiana* and in the natural host cassava. This shows that whilst generating ICs for CBSV is still a technical challenge, a structured approach, evaluating both in vitro and *in planta* transcription systems should successfully deliver ICs, allowing further study into the symptomology and virulence factors in this important disease complex.

**Electronic supplementary material:**

The online version of this article (10.1007/s12033-018-0139-7) contains supplementary material, which is available to authorized users.

## Introduction

Cassava brown steak disease (CBSD) is the most important disease threatening cassava (*Manihot esculenta*) production in Africa [1, 2]. CBSD is caused by at least two viral species: CBSV and UCBSV, which belong to the *Ipomovirus* genus of the *Potyviridae* family, hereafter referred to as U/CBSVs [3, 4]. The disease symptoms include brown necrotic lesions in the tuberous roots making them unfit for human consumption [5]. The disease was first reported in the 1930s in coastal regions of Tanzania [6] and until recently CBSD was largely restricted to lowland coastal areas of East Africa. However, in recent years CBSD has spread rapidly through Eastern and Central Africa, increasing in both geographical range and altitudinal distribution [1, 2]. CBSD is now the leading cause of cassava losses in East Africa and its on-going spread threatens the food security of subsistence farmers across sub-Saharan Africa [1].

Efforts to understand U/CBSV infection mechanisms, as well as testing varieties for resistance to these viruses, have been hampered by difficulties in accurately comparing infections. Transmission rates using the whitefly vector are low under laboratory conditions and to efficiently generate U/CBSV infections, plants are either mechanically inoculated with U/CBSV from infected plant material or grafted with U/CBSV infected buds. This often results in researchers using locally adapted, field-isolated viral inoculum, giving little consistency between different studies and making comparisons of infections difficult [7]. Viral populations can also change during *in planta* propagation further complicating comparisons between infections.

The development of infectious clones (ICs) for U/CBSVs would alleviate some of the difficulties encountered in current studies. ICs are stable cDNA copies of viral sequences, which can be used to consistently infect plants with the same defined viral genotype and thus enable reliable comparisons between infections. Furthermore, manipulations of U/CBSV ICs would enable studies to identify the functions of viral sequences, for instance chimeric ICs could be used to characterise viral sequences responsible for the differing symptoms caused by different CBSD viral isolates. Unfortunately, the construction of full-length U/CBSV ICs has been problematic for several reasons. Firstly, the genome size (> 9 kb) is too large for one-step reverse transcription (RT) and cloning of the single RT-PCR product encoding the viral genome [8]. Secondly, *Potyviridae* genome sequences are notoriously unstable during plasmid propagation in *Escherichia coli*, which may be due to the production of toxic proteins [9–11]. The resulting clones usually have sequence alterations such as construct rearrangement, accumulative point mutations, deletions and insertions. These alterations provide sequence stability and permissibility in *E. coli* yet render the IC non-infective or reduce its biological capabilities.

Several techniques have been employed to alleviate the toxicity issues of other *Potyviridae* ICs in *E. coli*. Plant derived introns may be inserted into viral sequences to disrupt the viral genome and thus prevent toxic protein expression in *E. coli*. ICs containing such introns can then be introduced into plants using *Agrobacterium tumefaciens*. Once the T-DNA with the IC constructs are inside plant cells, in vivo transcription coupled with intron splicing generates infectious viral transcripts [12]. While it is sometimes possible to produce stable intron-less infectious potyviral clones [8], multiple intron insertions may be necessary to successfully stabilise the viral sequences [12]. Rather than stabilising the full-length genome, an alternative approach is to maintain the genome as two separate stable clones, which can then be ligated together before in vitro transcription [10]. In addition, the viral genome can be modified through site directed mutagenesis of cryptic prokaryotic promoter elements to prevent the production of toxic proteins in *E. coli* [9]. However, this requires knowledge of such sequences and would result in an altered viral sequence.

This work evaluates the construction of CBSV and UCBSV ICs using a pipeline based on three methods:

Assembly of full-length UCBSV ‘Kikombe’ IC via assembly of PCR fragments in yeast, with stability in *E. coli*, in vitro transcription, followed by infection;

Assembly of full-length CBSV ‘Nampula’ IC as two genome halves due to sequence instability in *E. coli*, in vitro ligation followed by in vitro transcription, followed by infection;

Assembly of full-length CBSV ‘Tanza’ IC in *E. coli* with introns inserted for stability in *E. coli, in planta* delivery via *A. tumefaciens* infiltration for in vivo transcription and intron splicing.

Here we report that the methods resulted in usable constructs in *E. coli* and full-infectivity was confirmed through inoculation onto *Nicotiana benthamiana, N. clevelandii* and cassava. We propose a pipeline to produce ICs that tailors the construction method to the stability of the sequence. We believe the proposed methods can be applied to a wide range of viral sequences to produce ICs, which have diverse utility in the study of viral diseases.

## Materials and Methods

### Yeast-Based Plasmid Construction by Homologous Recombination

Plasmids were primarily constructed by yeast-based homologous recombination, using a series of overlapping fragments of viral genome derived by RT-PCR. Plasmid vectors (pYES2 or pCAMBIA0380) were linearized with appropriate restriction enzymes. Insert fragments were designed to contain at least 30 bp of homologous sequences to vector and/or neighbouring insert fragments according to the protocol outlined in Duff-Farrier et al. [13]. *S. cerevisiae* strain 10000 (Euroscarf) was transformed following the protocol from Gietz et al. [14] with 34 µl of mixed DNA fragments in appropriate concentrations (3:1 ratio of insert fragments: linearized vector backbone). The cells were resuspended then incubated at 30 °C for 30 min, followed by 42 °C for 30 min. Plasmids were rescued from yeast colonies using the Zymoprep Yeast Plasmid Miniprep II kit (Zymo Research, USA) and transformed into electrocompetent *E. coli* strains. Transformant *E. coli* colonies were cultured in Luria–Bertani (LB) broth containing 100 mg/ml ampicillin for pYES2 plasmids or 50 mg/ml kanamycin for pCAMBIA0380 plasmids at 37 °C at 220 rpm overnight and plasmids extracted using GeneJet Plasmid Miniprep Kit (ThermoFisher Scientific). Recombinant plasmids were analysed by restriction digest, PCR and sequence analysis to confirm correct construction.

### The Pipeline for IC Construction

**Fig. 1:**
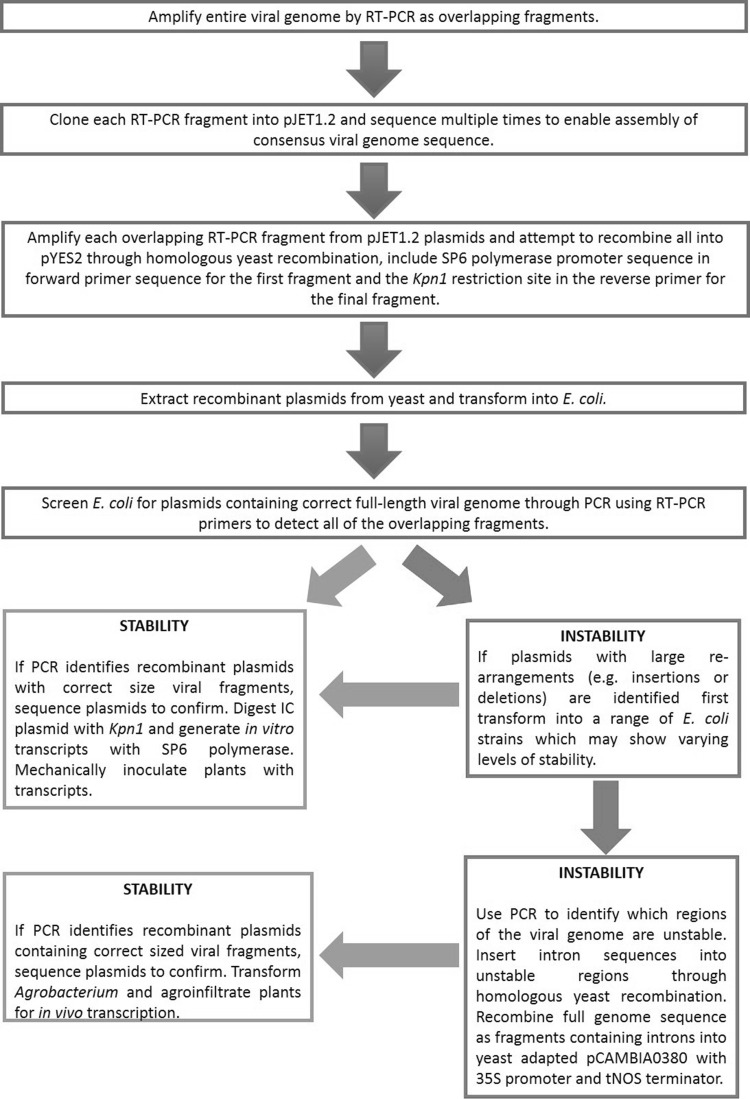
Recommended pipeline to overcome sequencing instability issues during the construction of *Potyviridae* ICs

The pathway for construction of ICs followed a logical progression of increasing complexity reflecting the level of sequence stability (Fig. 1). First, a simple in vitro transcription construct was attempted, containing a full-length viral genome to be transcribed via an SP6 promoter, and with a unique *Kpn*I site placed after the dA_18_ tail to prevent transcriptional run-through. Each viral genome was amplified by RT-PCR as a series of overlapping fragments of 1–2 kb, and these were recombined en-masse along with the SP6 promoter region into a linearised pYES2 yeast plasmid (see supplementary material for full details). If a direct one-pot recombination failed, a step-wise approach was used to progressively add each fragment in turn until the viral genome was complete. Where this proved unsuccessful, it served to highlight the viral regions that made the constructs unstable. The viral genome was split at the unstable position and cloned as two separate genomic regions that would be amenable to subsequent in vitro ligation and transcription.

Where it proved impossible to generate either a complete or split in vitro transcribed IC, an alternative strategy was deployed. The viral genome was assembled, again by yeast-based recombination, but in this instance cloned into a yeast-adapted version of the *Agrobacterium* vector pCAMBIA0380 to facilitate *in planta* transcription along with processing of inserted introns. The viral genome was recombined into the vector, along with a CaMV 35S promoter to drive transcription *in planta* and introns were built into the viral genome as detailed in the supplementary material to deliver stability to the plasmids for propagation in *E. coli*.

### Plant Cultivation

*N. benthamiana* and *N. clevelandii* plants were cultivated from seed produced in-house and planted in Sinclair all-purpose potting compost. They were grown in a controlled greenhouse with conditions of 25 °C with a 16 h/8 h: light/dark cycle. Infected plants were cultivated in growth cabinets 28 °C with a 16 h/8 h: light/dark cycle.

The CBSD susceptible cassava variety Colombia was propagated by planting vegetative stem cuttings in 16-cm pots containing multipurpose compost (William Sinclair) and maintained in a glasshouse 28 °C with a 16 h/8 h light/dark cycle. Various cassava infection methods were performed as described in the results.

### Mechanical Inoculation of Plants with Infectious Material

To deliver the full-length in vitro ICs, corresponding purified plasmid was linearized at the viral 3′ terminus using the restriction enzyme *Kpn*I. The split genome IC clones were digested with appropriate restriction enzymes, then ligated in vitro using T4 ligase to generate full-length templates prior to in vitro transcription as outlined below. Infectious transcripts were generated in vitro from the linearized templates [13, 15–17] using a Riboprobe in vitro SP6 polymerase transcription system (Promega), in conjunction with a Ribo-m^7^G Cap Analog (Promega). Plants were mechanically inoculated by dusting the first mature leaf from the growing tip with carborundum (particle size 180 grit) and gently stroking with the infectious plant material or viral transcripts that were suspended in phosphate inoculation buffer, according to Ogwok et al. [18].

### Agroinfiltration of Plants for In Vivo Transcription of Viral Transcripts

*Agrobacterium* infiltration of *Nicotiana* spp. was performed based on a modification of the method of Voinnet et al. [19]. Electrocompetent *A. tumefaciens* strain LBA4044 were transformed with the CBSV ‘Tanza’ IC plasmid (pCAM_CBSV_Tanza_Introns123) and transformed colonies were cultured in LB broth containing kanamycin 50 µg/ml and rifampicin 20 µg/ml for 48 h at 28 °C with 200 rpm. This culture was used to inoculate LB broth containing kanamycin 50 µg/ml, rifampicin 20 µg/ml, 10 mM Morpholino Ethane Sulfonic acid buffer (MES) and 150 µM of acetosyringone-freshly prepared in dimethyl sulfoxide (DMSO). After 20 h, the cells were pelleted at 6000 × g, washed with 10 mM MES and re-pelleted. The cells were then re-suspended in infiltration buffer (10 mM MgCl_2_; 10 mM MES and 150 µM acetosyringone-freshly prepared in DMSO) to an OD_600_ of between 0.5 and 0.7. The cells were incubated at room temperature for 3–6 h before being infiltrated into the leaves. Leaves were infiltrated through the stomata with 3 ml of *A. tumefaciens* suspension using a 10-ml syringe pressed against the lower leaf surface until soaking of the leaf was apparent.

Conventional agroinfiltration of cassava leaves proved to be impossible likely due to their hydrophobic surface and small sunken stomata, which effectively prevented the suspension from entering the leaf. The use of an abrasive with a surfactant has been shown to enhance transformation efficiency [20]. To overcome the difficulties in agroinfiltration of cassava, the surfactant Pluronic F-68 (Life Technologies) was added to the suspension at a concentration of 0.01% v/v [21] and mechanical inoculation of leaves with *A. tumefaciens* suspension soaking was tested. The *A. tumefaciens* culture was applied to the leaf surface of the first fully expanded leaf, along with carborundum powder and the leaf gently stroked to cause mechanical damage and allowing *Agrobacterium* access into wounded leaf cells.

## Results and Discussion

### Direct Generation of an In Vitro UCBSV ‘Kikombe’ Infectious Clone

The full-length UCBSV ‘Kikombe’ genome could be assembled and stably propagated in *E. coli* strain α-select without the need for any sequence modifications. The full-length sequence for UCBSV ‘Kikombe’ was amplified in eight fragments and cloned under control of the SP6 promoter for in vitro transcription in the pYES2 plasmid by yeast recombination. Restriction digestion, PCR and sequence analysis confirmed the integrity of the UCBSV ‘Kikombe’ IC sequence (NC_KX753357.1) with no obvious rearrangements. High plasmid yield could only be obtained if freshly transformed cells were used and the plasmid recovered immediately from fresh colonies. High amounts of plasmids could also be obtained by performing large scale plasmid extraction using the ThermoFisher Scientific pJET Midi Prep Plasmid Extraction Kit. Isolated plasmids were used as template for in vitro synthesis of viral transcripts for infection.

### Infections of *Nicotiana* spp. with UCBSV ‘Kikombe’ IC

**Fig. 2:**
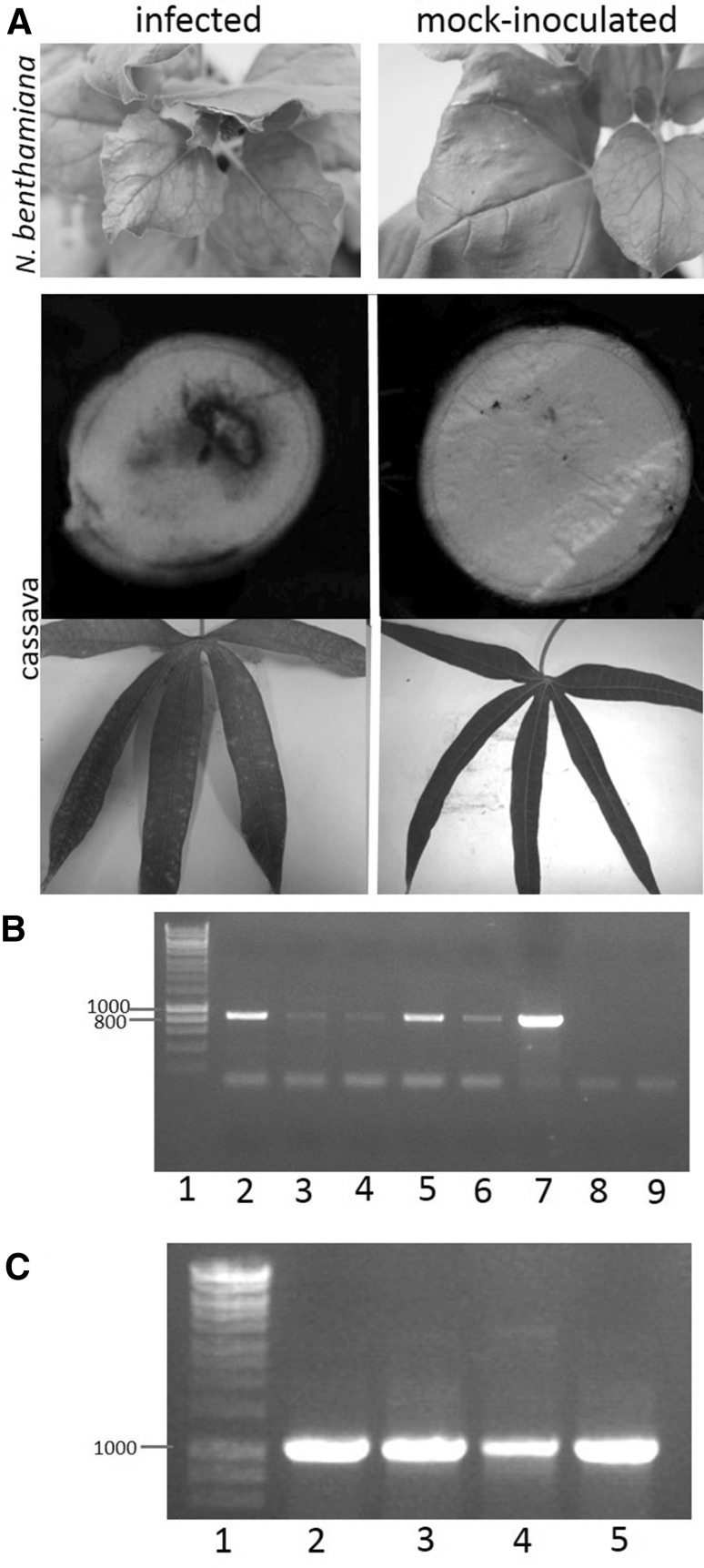
Infection of *N. benthamiana* and cassava with UCBSV ‘Kikombe’ IC. **a** Disease symptoms on leaves of infected *N. benthamiana* and cassava and tuberous roots of infected cassava include: mild foliar mosaic pattern in systemic leaves at 21 days post inoculation (dpi) with UCBSV ‘Kikombe’ IC infectious transcripts compared to healthy leaves of mock-inoculated plant; mild foliar chlorosis in cassava plant infected with UCBSV ‘Kikombe’ IC at 6 weeks post infection compared to healthy leaves of a mock-inoculated plant; and, tuber necrosis in UCBSV ‘Kikombe’ IC infected plant at 12 months post infection compared to the healthy tuber of a mock-inoculated at 12 months post mock inoculation. **b** RT-PCR detection of UCBSV ‘Kikombe’ in *N. benthamiana* and *N. clevelandii*. Primers amplified the 867 bp RT-PCR product of the UCBSV CI region from an infected *N. benthamiana* plant (lane 2) and two infected *N. clevelandii* plants (lanes 5 and 6) at 14 dpi. RT-PCR amplification from a *N. benthamiana* inoculated with infectious material containing the wild-type virus confirm correct amplicon size. No amplification appears in the mock-inoculated plant (lane 8) or no template control (lane 9). The 1 Kb Hyperladder (Bioline) is shown in lane 1. **c** Primers amplified the 1 Kb RT-PCR product targeting the UCBSV Vpg—NIa region from four IC-infected cassava plants 42 dpi (lanes 2–5). No amplification occurred in from the mock-inoculated plant or in the no template control (data not shown). The 1 Kb Hyperladder (Bioline) is shown in lane 1

To infect indicator plants, capped in vitro transcripts were generated from the UCBSV ‘Kikombe’ IC and mechanically inoculated onto *N. benthamiana* and *N. clevelandii*. At 21 days post inoculation (dpi) all ten *N. benthamiana* displayed systemic mild mosaics (Fig. 2), while *N. clevelandii* was asymptomatic (data not shown). The timing and symptomatology of the IC infections were the same as observed when plants were infected with the wild-type UCBSV ‘Kikombe’ inoculum. RT-PCR detected UCBSV in systemic leaves at 14 dpi (Fig. 2). The resulting RT-PCR products were cloned and sequenced, which confirmed the presence of UCBSV IC sequence with expected IC SNPs in both *N. benthamiana* and *N. clevelandii*.

To test the transmissibility of the IC infections, systemic leaves from two UCBSV IC infected *N. benthamiana* plants were mechanically back inoculated onto ten healthy *N. benthamiana* plants. UCBSV symptoms developed in 50% of tester plants and UCBSV infection was detected in all plants by RT-PCR.

To test infectivity in cassava, four plants were mechanically inoculated with infectious UCBSV ‘Kikombe’ IC in vitro transcripts. At 42 dpi, UCBSV IC sequence was detected in the upper systemic leaves of all four inoculated plants through RT-PCR and amplicon sequencing identified the expected IC SNPs to indicate infection was generated from the IC (Fig. 2). After 8 weeks post infection, these plants also displayed mild foliar chlorosis (Fig. 2). Systemic cassava leaf material in IC infections was used to mechanically back-inoculate *N. benthamiana* and *N. clevelandii*. UCBSV IC sequence was detected in systemic leaves of both plant species by RT-PCR. At 12 months post infection, tubers from cassava plants inoculated with UCBSV IC transcripts showed necrotic patches, whereas no necrosis had developed in the tubers of mock-inoculated plants (Fig. 2) and the presence of UCBSV was detected by RT-PCR on symptomatic tuber material. These experiments demonstrate that the UCBSV ‘Kikombe’ IC is infectious to *N. benthamiana, N. clevelandii* and cassava hosts and infections generate symptoms that are indistinguishable from those during infections with the wild-type UCBSV inoculum.

### Ligation of Two Halves of the CBSV ‘Nampula’ Genome, Followed by In Vitro Transcription

Following unsuccessful attempts to generate a full-length infectious clone for CBSV ‘Nampula’, sequential cloning of each fragment identified fragment three containing the CI region of the genome as unstable. It has been suggested that sequence instability is due to the expression of toxic proteins from cryptic prokaryotic promoter elements [9, 22]. It is possible that the less stable CBSV CI region contains prokaryotic promoters that are more efficient at promoting toxic protein expression. To overcome this instability, the genome was cloned in two fragments splitting the CI containing fragment at a unique *Psy*I restriction site (Fig. S2). These two fragments could be stably produced in *E. coli*. The two fragments were ligated together at the *Psy*I restriction site using T4 DNA ligase. The full-length ligated sequence was used as a template for in vitro SP6 polymerase transcription to generate infectious transcripts for mechanical inoculation of plants.

### CBSV ‘Nampula’ IC Infections

**Fig. 3:**
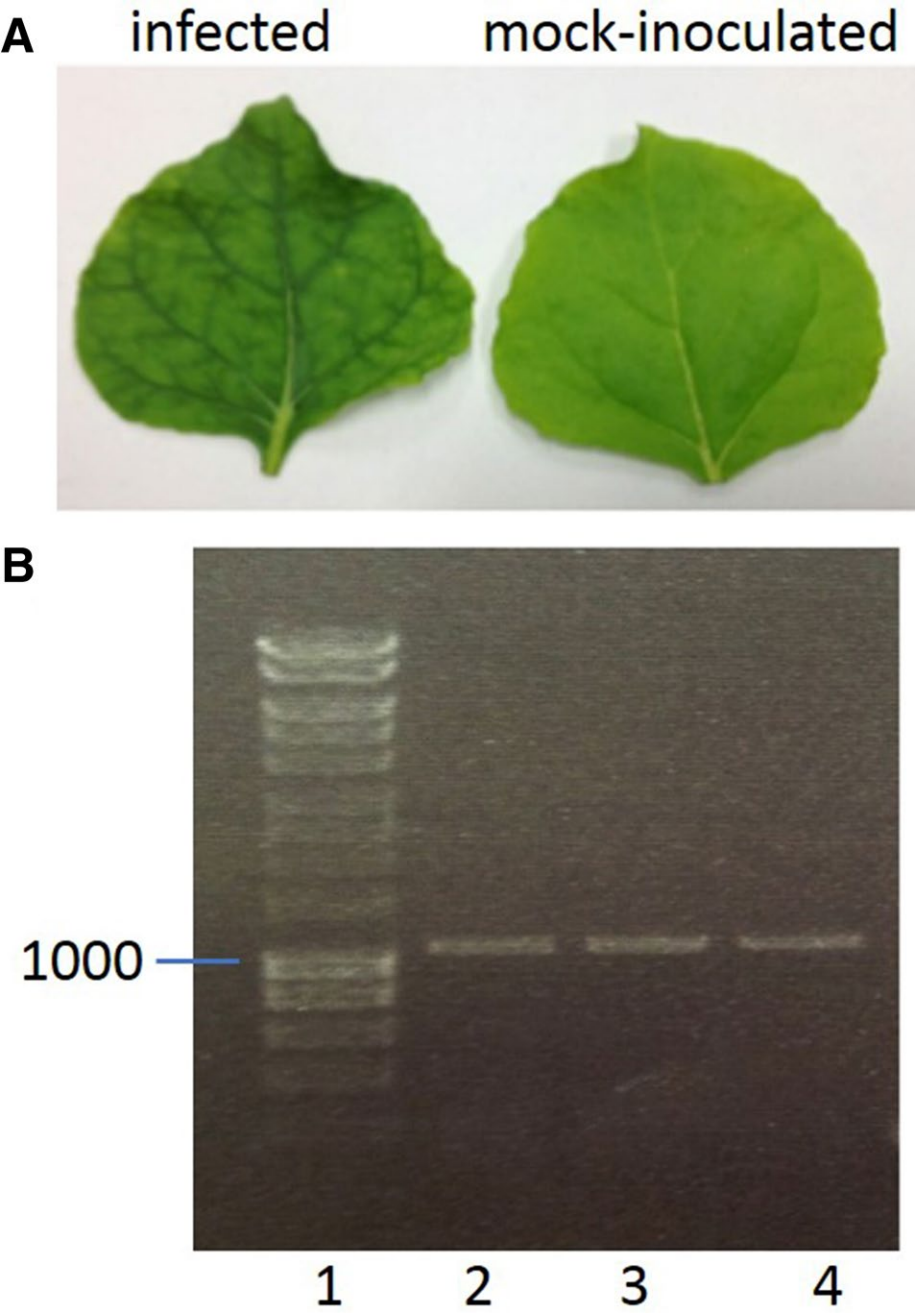
CBSV ‘Nampula’ infections of *N. benthamiana*. **a** Mild systemic foliar mosaic pattern at 14 dpi with CBSV ‘Nampula’ IC compared to healthy leaves of mock-inoculated plant. **b** RT-PCR detection of CBSV ‘Nampula’ IC *N. benthamiana* infections amplified the 1 Kb RT-PCR product targeting the CBSV ‘Nampula’ coat protein in three *N. benthamiana* plants infected with the CBSV ‘Nampula’ IC at 14 dpi (lanes 2–4). No amplification occurred from the mock-inoculated plant or no template control. The 1 Kb Hyperladder (Bioline) is shown in lane 1

In vitro transcription was performed directly from the ligation mixture of the bipartite CBSV ‘Nampula’ clones and the resulting reaction was mechanically inoculated onto *N. benthamiana* and *N. clevelandii*. At 14 dpi *N. benthamiana* displayed light systemic mosaic symptoms (Fig. 3), while *N. clevelandii* was asymptomatic (data not shown). Successful infection of both *N. benthamiana* and *N. clevelandii* infections was confirmed by RT-PCR and sequencing of RT-PCR products (Fig. 3). The low rate of infection with the CBSV ‘Nampula’ IC may be due to low plasmid concentration, resulting in a reduced number of ligated genomes and template for in vitro transcription. Due to low infection rates of *Nicotiana* spp., no attempts to infect cassava with viral transcripts were made.

### Intron Insertion and Construction of an *Agrobacterium* Expression Cassette for the CBSV ‘Tanza’ Isolate

Initial attempts to construct a full-length CBSV ‘Tanza’ IC for in vitro transcription were unsuccessful due to sequence instability in *E. coli*. Introns were inserted into the unstable regions P3, CI and NIb. The complete genome with inserted introns had sufficient sequence stability in *E. coli* strain OverExpress C43(DE3).

To enable the IC sequence to be delivered into plant cells for in vivo transcription and removal of introns, the sequence was cloned into plant expression vector pCAMBIA0380 downstream of the CaMV 35S promoter [23] and upstream to the tNOS terminator. Restriction digest, PCR and sequence analysis confirmed successful construction of the full-length CBSV ‘Tanza’ IC (NCBI: MG570022). Once stability was confirmed the plasmid was moved to *Agrobacterium tumefaciens* for subsequent infection of plants.

### CBSV ‘Tanza’ IC Infection of *Nicotiana* spp.

The CBSV ‘Tanza’ IC plasmid was transformed into *A. tumefaciens* and agroinfiltrated into the indicator plants: *N. benthamiana* and *N. clevelandii*. At 7 dpi *N. benthamiana* developed localised necrotic lesions on the infiltrated leaves. After 8 dpi, vein necrosis was observed on systemic, emerging leaves, which went onto curl and display severe necrosis at 8–12 dpi (Fig. 3). Necrosis also developed in agroinfiltrated *N. clevelandii* leaves at 7 dpi and systemic leaves at 10 dpi (Fig. 3). Buffer inoculated mock controls developed no symptoms.

Successful infection of *N. benthamiana* and *N. clevelandii* plants agroinfiltrated with the CBSV ‘Tanza’ IC was confirmed by RT-PCR, targeting the NIb region containing intron 3. Amplification of the 500 bp product demonstrates successful infections of all agroinfiltrated plants and was consistent with splicing out of intron 3 (Fig. 3). *N. benthamiana* infections with the CBSV ‘Tanza’ IC developed severe necrosis earlier in infection than wild-type CBSV ‘Tanza’ infections. It was found that agroinfiltration was efficient at generating infection in *N. benthamiana* with all plants (*n* = 10) developing infections, whereas only 30% of *N. benthamiana* plants mechanically inoculated with infectious material containing wild-type CBSV ‘Tanza’ developed infections (*n* = 10).

### CBSV ‘Tanza’ IC Infection of Cassava

**Fig. 4:**
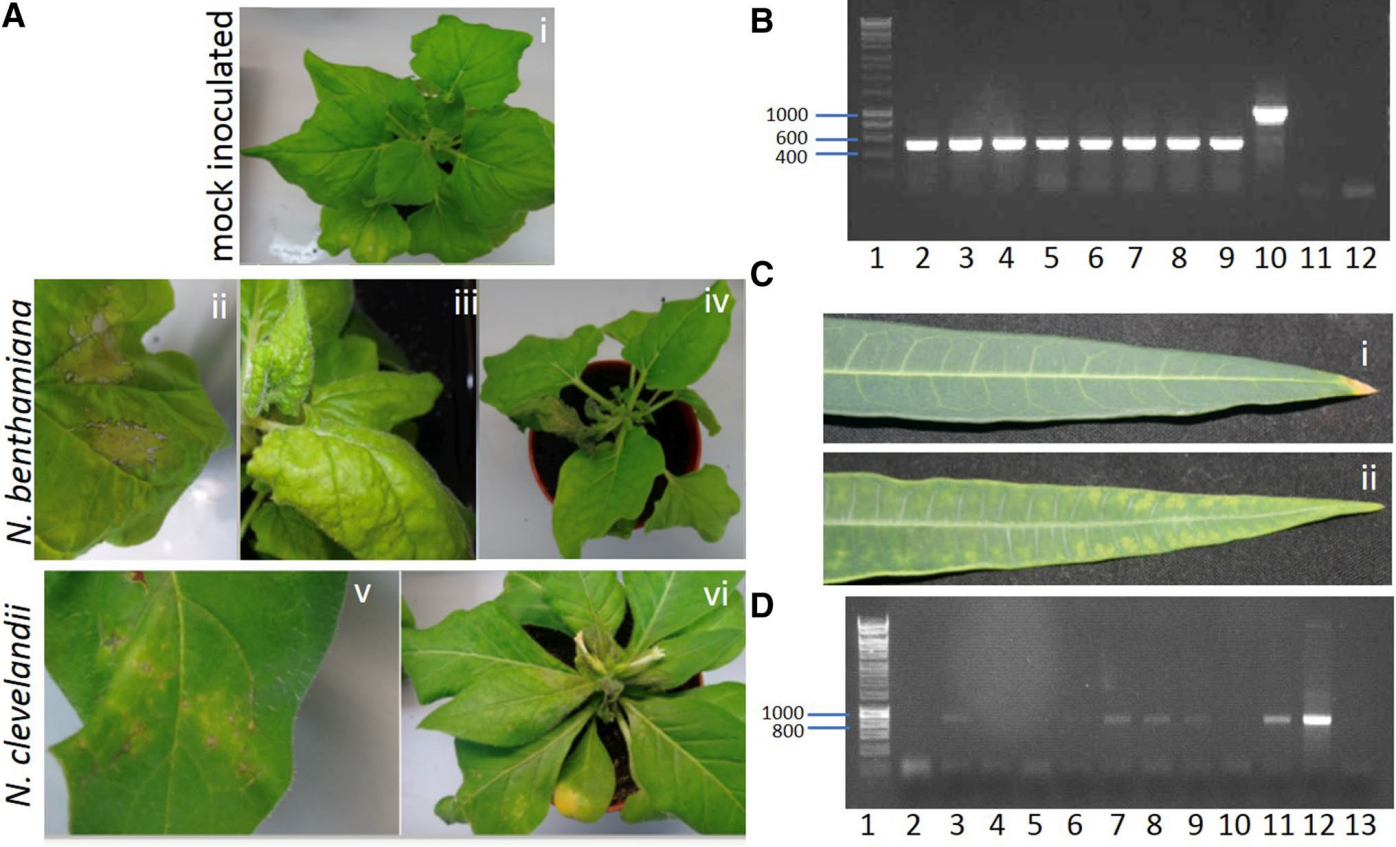
Infection of *N. benthamiana, N. clevelandii* and cassava with CBSV ‘Tanza’ IC. **a** Disease symptoms on *N. benthamiana* following infection with CBSV ‘Tanza’ IC include strong chlorosis and necrosis on the leaf agroinfiltrated with the IC (ii), leaf curling and chlorosis of upper systemic leaves (iii), and systemic wilting and necrosis (iv) at 12 dpi compared to healthy mock-inoculated plant (i). Disease symptoms on *N. clevelandii* include chlorosis and necrosis on the agroinfiltrated leaf at 12 dpi (v) and necrosis of upper systemic leaves at 12 dpi (vi). No necrosis was observed in the mock-inoculated plant (data not shown). **b** Detection of CBSV ‘Tanza’ in infected *N. benthamiana* and *N. clevelandii* by RT-PCR. Amplification of 500 bp of CBSV NIb sequence from four *N. benthamiana* plants (lanes 2–5) and four *N. clevelandii* (lanes 6–9) agroinfiltrated with the IC at 14 dpi indicating successful infection and splicing of intron 3. Amplification of 1 Kb product from pCAM_CBSV_Tanza_Introns123 plasmid is shown in lane 8 and no amplification occurred from mock-inoculated plant (lane 11) or no template control (lane 12). The 1 Kb Hyperladder (Bioline) is shown in lane 1. **c** Chlorosis on a systemic leaf at 42 dpi on infected (ii) cassava compared to symptomless mock-inoculated (i) cassava. **d** RT-PCR amplified the 700 bp RT-PCR product targeting the CBSV ‘Tanza’ NIa region in one of four cassava plants agroinfiltrated in the first experiment (lane 3) and three of four cassava plants agroinfiltrated in the second experiment at 42 dpi (lanes 7, 8 and 9). Amplification is shown in positive controls: *N. benthamiana* agroinfiltrated with the IC (lane 11) and pCAM_CBSV_Tanza_Introns123 template (lane 12). No amplification occurred in negative controls: from a mock-inoculated plant (lane 10) or no template control (lane 13). The 1 Kb Hyperladder (Bioline) is shown in lane 1

Although conventional agroinfiltration failed to infect cassava with the CBSV ‘Tanza’ IC, a modified method for infection of cassava by agroinfiltration with the aid of carborundum powder and the surfactant Pluronic F-68 (Life Technologies) resulted in successful infection of cassava via *Agrobacterium* inoculation. CBSV infection was detected by RT-PCR in two out of three cassava plants in the first experiment and three out of three plants in the second experiment (Fig. 4). Cassava leaves from agroinfiltrated plants showing chlorosis (Fig. 4) were taken and used to mechanically back-inoculate three *N. benthamiana* plants. Two of these plants developed mild systemic leaf curling at 14 dpi and the CBSV ‘Tanza’ sequence was detected in these symptomatic plants by RT-PCR and amplicon sequencing.

## Conclusions

This work has demonstrated that it remains a technical challenge to generate viable ICs for the U/CBSV virus complex. Like Pasin et al [24], we were able to generate a successful IC for UCBSV by direct cloning. However, this ‘Kikombe’ clone was not stable for subsequent modifications, therefore limiting the utility of the approach and suggesting that UCBSV ICs may not all display stability through a direct cloning approach. Attempts to generate ICs for the two CBSV isolates, ‘Nampula’ or ‘Tanza’, each resulted in unstable plasmids or plasmids with significant rearrangements and/or deletions. Whilst the stability problems with CBSV ‘Nampula’ sequences were overcome by splitting the CI region over two plasmids in *E. coli*, additional regions of the CBSV ‘Tanza’ genome were also found to be unstable in *E. coli*, which precluded such an approach and required the insertion of three introns to stabilise the genome. The three viral genome sequences therefore displayed different levels of sequence stability, which could only be determined on a case by case basis. Such cloning issues have also been encountered when cloning other *Potyviridae* viruses [8, 25, 26]. In addition to the approaches presented here, unstable propagation of *Potyviridae* virus ICs in *E. coli* has been overcome by direct movement of the IC into *A. tumefaciens* [24, 27]. This approach may present its own set of difficulties including the lower transformation efficiency of *A. tumefaciens*.

Here, we have highlighted some of the technical challenges presented by these viruses, both in the generation of stable ICs and in the ways that these may then be introduced into their hosts. Each of the approaches has its own merits and problems, both in terms of the complexity of the constructs and the ease of their application. In addition, we note that even when the initial IC is stable, it may not prove to be stable when used for further modification. We provide our recommendations for the construction of full-length *Potyviridae* ICs in the pipeline in Fig. 1.

Given that different isolates of CBSV and UCBSV can result in very different symptoms and severity on different cultivars, both in terms of foliar and tuber impacts, the construction of chimeric ICs will enable the identification of viral sequences responsible for differential symptom development and host interactions. This will require the construction of clones that are stable following further manipulation. Where the screening of CBSD-resistant transgenic cassava lines has so far been hampered by poor whitefly transmission in the laboratory, the potential deployment of these ICs will allow for effective reliable transmission and therefore support progress in this area. This work represents a crucial milestone in the fight against CBSD and increasing food security for Africa.

## Electronic supplementary material

Below is the link to the electronic supplementary material.

Supplementary material 1 (DOCX 719 KB)
