# Supplementary material for: Strategies for the Construction of Cassava Brown Streak Disease Viral Infectious Clones
Source: Mol Biotechnol. 2018 Nov 27;61(2):93–101. doi: 10.1007/s12033-018-0139-7 (PMC6513833; doi:10.1007/s12033-018-0139-7)
Supplement: Supplementary file 1 — Supplementary material 1 (DOCX 719 KB) [file 12033_2018_139_MOESM1_ESM.docx]

## Generation of a full length UCBSV ‘Kikombe’ infectious clone by direct recombination and *in vitro* transcription

As outlined in Figure S1, RNA was extracted using a modified CTAB method (13) from symptomatic cassava collected from Mikocheni Road, Tanzania provided as frozen leaf material courtesy of the Natural Resources Institute (NRI). First strand cDNA was synthesised using a RevertAid First Strand cDNA Synthesis Kit (ThermoFisher Scientific) primed with oligo(dT)_18_, according to the manufacturer’s protocol. To assemble the UCBSV ‘Kikombe’ genome sequence, degenerative primers amplified eight overlapping fragments using conserved sequences within UCBSV genomes available in the NCBI database using High-fidelity Phusion (ThermoFisher Scientific)(Table S1). To amplify Fragment 4, a gene specific primer was required instead of oligo(dT)_18_ for priming of cDNA synthesis. Each fragment was cloned into pJET1.2 and sequenced five times to generate consensus sequences. The consensus sequences were used to assemble the UCBSV ‘Kikombe’ genome sequence (NC_KX753356.1). This sequence was aligned to the sequence of the UCBSV ‘Namulonge’ isolate (NC_014791.1) and showed 92% nucleotide sequence identity.

Using the assembled UCBSV ‘Kikombe’ genome sequence it was then possible to design specific UCBSV ‘Kikombe’ specific primers U1 – U16 (Table S2) to amplify 8 fragments encompassing the entire genome. Fragments 1, 2, 3 and 4 and 5, 6, 7 and 8 were recombined by yeast recombination into separate pYES2 plasmids to form pYES2_Kik_1234 and pYES2_Kik_5678 (Fig. S2). To enable *in vitro* transcription the SP6 polymerase promoter sequence was inserted at the 5’ end of the genome through PCR amplification of fragment 1234 with the forward primer U21 containing the SP6 promoter sequence. To generate a linear template for *in vitro* transcription, the KpnI restriction site was inserted after polyA tail through PCR amplification of fragments 5678 with a reverse primer U20 containing the KpnI restriction site sequence. Fragments 1234 and 5678 were amplified from their respective pYES2 plasmids and recombined together into pYES2 to form pYES2_UCBSV_Kik_Full through homologous yeast recombination (Fig. S1).

The *E. coli* strain used for plasmid propagation has been shown to influence stability of *Potyviridae* ICs (12) and therefore we tested several strains (Sure 2 [Agilent Technologies], CD4, Able K [Agilent Tecnologies], XL10-Gold [Agilent Technologies], DH5α, and α-select [Bioline] cells) for optimal plasmid yield. *E. coli* strain α-select (Bioline) produced the highest plasmid yields. Purified plasmid was linearized at the viral 3′ terminus using the KpnI restriction enzyme. The linearized plasmid was purified using the GeneJet Gel Extraction kit (ThermoFisher Scientific). Infectious transcripts were generated *in vitro* from the linearized templates (14-17) using a Riboprobe *in vitro* SP6 polymerase transcription system (Promega), in conjunction with a Ribo-m^7^G Cap Analog (Promega). Plants were mechanically inoculated with the infectious transcripts. Successful infection was determined by RT-PCR with the following primers: Vpg – NIa forward (5′ ATTGARCCAACCACRAGYGGACCYATG 3′) and Vpg – NIa reverse (5′ ACTGACCCTATATCYACATCATCAAC 3′); CI forward (5′GATTTTGGYGAACGCATA AGTCCAAA 3′) and CI reverse: (5′ CTCCATTTAAYTGGTTCAAGAGYAACTC 3′).

## Generation of CBSV ‘Nampula’ infectious clone as a two-piece system for ligation and *in vitro* transcription

As outlined in Figure S2, RNA was extracted from freeze-dried *N. benthamiana* infected with the CBSV ‘Nampula’ isolate from Mozambique courtesy of Fera (York, UK) with the Plant RNA Mini Kit (Omega Bio-tek) according to the manufacturer’s protocol. First strand cDNA was synthesised as described. Six overlapping RT-PCR fragments were amplified using primers designed based on the full length ‘Nampula’ genome sequence NC_MG019914 provided by Fera (York, UK) with High-fidelity Phusion (ThermoFisher Scientific) to minimise PCR-errors (Table S2). The RT-PCR fragments were cloned into pJET1.2 and sequenced three times. The assembled consensus sequence NC_MG019915 showed 99% nucleotide sequence identity with NC_MG019914, confirming the origin of the infectious material used.

Initial attempts to assemble an intron-less IC of the CBSV ‘Nampula’ isolate proved unsuccessful due to sequence instability in *E. coli* with large deletions detected between fragment 1 and fragment 5. To identify the unstable region, fragments were sequentially cloned and clones containing fragment 3 were identified as unstable. Fragment 3 containing the CI region of the genome was digested at a unique PsyI restriction site to generate two fragments (fragments 3a and 3b) (Fig. S2). The 5′ half of the viral genome consisting of fragments 1, 2 and 3a were recombined by yeast recombination in to the pYES2 vector to form pYES2_Nam_123a. Primer N13 containing the SP6 promoter sequence was used to amplify F1. The 3′ half of viral genome consisting of fragment 3b, 4, 5 and 6 was recombined by yeast recombination to form pYES2_Nam_3b456. Fragment 123a was then liberated from pYES2Nam_123a through restriction digest with HpaI and PsyI and fragment 3b456 was liberated from pYES2Nam_3b456 through restriction digest with PsyI and KpnI. The two fragments were ligated together at the PsyI restriction site using T4 DNA ligation Kit (NEB Biolabs)(Figure S2). The full-length ligated sequence was used as a template for *in vitro* transcription as described for the UCBSV ‘Kikombe’ IC. Infectious transcripts were used to mechanically inoculate plants. Successful infection was determined by RT-PCR with primers designed to amplify the sequence encoding the coat protein (forward: 5′ ATTTACAAAAAAGCAGGCTCCGCGGCCGCCATGGCAATTGACAAGGATGAGATTGAAG 3′ and reverse: 5′ ACTAATGCCAACTTTGTACAAGAAAGCTGGGTCTTATTCAATAGCGGCACCCGCGTAG 3′).

## Generation of a CBSV ‘Tanza’ infectious clone as an *in planta* transcribed IC delivered by Agroinfiltration

As illustrated in Figure S3, frozen leaf material from symptomatic cassava along Mikocheni Road, Tanzania infected with the CBSV ‘Tanza’ isolate courtesy of the Natural Resources Institute (NRI) was mechanically inoculated onto *N. benthamiana*. RNA was extracted from infected *N. benthamiana* plants as described and cDNA was synthesised using oligo(dT)_18_ primer. Primers were designed based on conserved sequences within CBSV genomes available in the NCBI database. The 5′ UTR sequence was amplified using the 5′ RACE System (version 2.0 Invitrogen). Each RT-PCR fragment was cloned into pJET1.2 and two amplicons were sequenced. The sequences were used to assemble the CBSV ‘Tanza’ genome NCBI_MF975780. Attempts to produce a stable complete genome failed due to instability in *E. coli*. To overcome this instability, the genome was first cloned as two halves that could be stably propagated in *E. coli*. Fragments 1, 2 and 3 were recombined into the pYES2 vector to generate pYES2_Tan_123 and fragments 4, 5 and 6 were recombined to generate pYES2_Tan_456 (Fig. S3). Introns were then inserted into the unstable regions P3, CI and NIb. Positions for intron insertion were selected from previous studies that identified regions of instability in *Potyviridae* ICs in *E. coli* (10, 12).

Intron sequences were amplified from the Pea seedborne mosaic virus infectious clone containing the corresponding intron sequences (12). The sequence for intron 1 (188 bp) originates from the *Solanum tuberosum* ST-LS1 gene, while intron 2 (220 bp) and intron 3 (508 bp) are from the *Phaseolus vulgaris* NiR gene (12). Introns were amplified with primers listed in Table S2: intron1 (T13 – T14), intron 2 (T15 – T16) and intron 3 (T17 – T18). Introns 1 and 2 were successfully inserted into P3 and CI regions of pYES2_Tan_123 and intron 3 was inserted into the NIb region of pYES2_Tan_456 through yeast recombination. The two halves of the viral genome, including introns were amplified by PCR from the respective plasmids and recombined into pYES2 to form pYES2_CBSV_Tanza_Introns, which demonstrated sufficient sequence stability in *E. coli*.

To enable the IC sequence to be delivered into plant cells for *in vivo* transcription and removal of introns, the viral sequence was cloned into the *Agrobacterium*-compatible vector with an expression cassette, pCAMBIA0380, which contains tNOS terminator. The full-length CBSV ‘Tanza’ sequence with introns was liberated from the pYES2 vector by restriction digest with KpnI and HindIII and recombined, along with the CaMV 35S promoter (amplified from pCAMBIA1300 with primers T19 and T20), into a yeast-adapted version of pCAMBIA0380 (18) to form pCAM_CBSV_Tanza_Introns123. Rescue into *E. coli* strain OverExpress C43(DE3) was sufficiently stable. Restriction digest, PCR and sequence analysis confirmed successful construction of the full-length CBSV ‘Tanza’ IC (NCBI: MG570022).

**Table S1.** Degenerate primers used to amplify UCBSV ‘Kikombe’ genome

| Primer ID | Sequence |
| --- | --- |
| CBSV2F  CBSV2R1 | 5’AAA TAA ACA TGA CAT AAG AAT AC3’  5’ CAT ATG TCA TGA TTG TAA TAG 3’ |
| GAP 5’ F  GAP5’R | 5’GAG TGC GAT GAC ATG TTT GA 3’  5’ ATC AAC ATC TCT ATT CCC GGT TTG 3’ |
| 5’ 1KB FWD  5’1KB REV | 5’ ATT GAR CCA ACC ACR AGY GGA CCY ATG 3’  5’ ACT GAC CCT ATA TCY ACA TCA TCA AC 3’ |
| 5’867bp F  5’ 867bp R | 5’GAT TTT GGY GAA CGC ATA AGT CCA AA 3’  5’ CTC CAT TTA AYT GGT TCA AGA GYA ACT C 3’ |
| 5’1125bpF  5’1125bp R | 5’ GCT GAG TGT GTB AAA GCT GCA AAG CC 3’  5’TTG TTR RCT CTG GYT TWG CRA CYT GAA CTT C3’ |
| 5’MID1KBF  5’MID 1KB R | 5’ GTA GCT CAT AAA ATA AGT CCA AA 3’  5’ CCA AGR YGY GCY CTR CCA AGT CTA ACC3’ |
| MID FF 1KB  MID RR 1KB | 5’ GCS CCA ATG GGA TTY CCA GAA GAA GAG GG 3’  5’ CAC TGA YCC AAC TCG AAC WGG CTC 3’ |
| 3’MID 1.5KB  3’MID 1.5KB | 5’ GGA CAT TYA ATC CTG ATC TTG TTG ART GG 3’  5’ CCT TCY ACA CGC TTT CTA TCC 3’ |
| 3’1.4KB F  3’1.4KB R | 5’ GGG CTR CCG RRA AAG GWT GTG 3’  5’ CCT GTA TAT TAA CTC CAT ATG CTT TAG C 3’ |
| 922bpFS  3’ Final | GGGCAATTGAGGTTGCCAAAGC  TTTTTTTTTTTTTTTTTTTTTGGAC |

**Table S2**. primers used for the construction of CBSD ICs. Homologous overlapping sequences for yeast recombination are underlined. The KpnI restriction enzyme recognition sequence is shown in bold. The SP6 promoter sequence is shown in blue. Genomic positions relative to the start codon are given as well as genomic positions for intron insertion.

| Primer ID (genome position) | Sequence |
| --- | --- |
| U1 (c. -132 – -110) | AAATAAACATGACATAAGAATAC |
| U2 (c. 2531-2551) | CATATGTCATGATTGTAATTG |
| U3 (c. 2248-2274) | ATTGAGCCAACTACGAGTGGACCTATG |
| U4 (c. 3293-3317) | ACTGACCCTATATCTACATCATCAAC |
| U5 (c. 3061-3086) | GATTTTGGTGAACGCATAAGTCCAAA |
| U6 (c. 3901-3928) | GAGTTACTCTTGAATCAATTAAATGGAG |
| U7 (c. 3517-3536) | AAAGAATACATTAGAATCGA |
| U8 (c. 4626-4649) | TCTTCTTCTGGAAATCCCATTGGA |
| U9 (c. 4626-4649) | TCCAATGGGATTTCCAGAAGAAGA |
| U10 (c. 5641-5664) | CACTAACCCAACTTGAACTGGCTC |
| U11 (c.5327-5355) | GGACGTTTAATCCTGATCTTGTTGAGTGG |
| U12 (c.6798-6818) | TCCAGCAATGGATACTCACAA |
| U13 (c. 6782-6802) | GGGCTGCCGAAAAAGGTTGTG |
| U14 (c.8170-8197) | GCCAAAGCATATGGAGTTAATATACAGG |
| U15 (c. 8156-8177) | GGGCAATTGAGGTTGCCAAAGC |
| U16 (c. 8933-8957) | TTTTTTTTTTTTTTTTTTTTTGGAC |
| U17 | GAGCGGCCGCCAGTGTGATGGATATCTGCAAAAAATAAACATGACATAAGAATAC |
| U18 | CTCACTATAGGGAATATTAAGCTTGGTACCAATTGGTACCACGCGGGATTAGTTGGCTCTGGTTTTGCAACCTGAACTTC |
| U19 | GAGCGGCCGCCAGTGTGATGGATATCTGCGCTCCAATGGGATTTCCAGAAGAAGA |
| U20 | CTCACTATAGGGAATATTAAGCTTGGTACCAATTGGTACCACGCG TTTTTTTTTTTTTTTTTTTTTGGAC |
| U21 | GAGCGGCCGCCAGTGTGATGGATATCTGCAAATTAGATCTATTTAGGTGACACTATGAAAAATAAACATGACATAAGAATAC |
| U22 | CTCACTATAGGGAATATTAAGCTTGGTACCGAATTGGTACCACGCG TTTTTTTTTTTTTTTTTTTTTGGAC |
| N1 (c. -127— -98) | AAATTTAAACTATGACATAAGAAAACATAA |
| N2 (c.946-975) | GAACATATCGTGCTCATTTTCCTGGACACT |
| N3 (c. 906-935) | GGTGAGACCGGGTTGGAGTGGTGCTGTTAT |
| N4 (c.2916-2945) | CTCCCGTCCACTTTAAGAACAGGTAAGCCT |
| N5 (c. 2881-2905) | GATGTTGATACGTGTGCTGAAAAGT |
| N6 (c.4876-4905) | CCCGCGAGTAGTTGATATCACGAGCGATTC |
| N7 (c. 4840-4864) | ATACCCTATCATTTGGCAGAGAATG |
| N8 (c.6876-6905) | GGTGGTAAGATTCCACTCAGCCATTCCTCG |
| N9 (c. 6836-6865) | GGGTTGAGGGGCTTTACTTGGATGAATTCA |
| N10 (c. 7950-7979) | CCACCACCAGCTTTTATTTTGAATCTTGTC |
| N11 (c. 7880-7904) | CTATGGAGCCACCTGCTGAACGAAG |
| N12 (c.8845-8883) | GTTACTTTTATATATTGTGTTACCACACTAAATAAAAGG |
| N13 | GAGCGGCCGCCAGTGTGATGGATATCTGCAATTTAGGTGACACTATAGAAATTTAAACTATGACATAAGAAAACATAA |
| N14 | GACATAACTAATTACATGATGCGGCCCTCTAGATGGAGCACTCCCAGGAATAAAAGTTTGATGAG |
| N15 | CTACTAGCAGCTGTAATACGACTCACTATAGGGAATATTACTCATCAAGTATTGCTTCAGTCCTTTCAGC |
| N16 | CTCACTATAGGGAATATTAAGCTTGGTACCGAATT**GGTACC**GTTACTTTTATATATTGTGTTACCACACTA |
| T1 (c. -127— -88) | AAATTTAAACTATGACATAAGAAAACATAAGAATACATTA |
| T2 (c.1359-1385) | GACTGGTCAGTGATGTGATCTTGTATC |
| T3 (c. 1087-1112) | TCATTTGACTTGAGTTGGGCACGGTC |
| T4 (c. 2965-2982) | AACCTGTCGTTGGACATC |
| T5 (c. 2115-2134) | TGAGAGGCAAAGCGGTGATG |
| T6 (c. 3979-4032) | AATATAGTCGCTAGCACATTGAGCCTCCAAGTACTCTGCTGAGTGCGGATTTTC |
| T7 (c. 4003-4024) | TTGGAGGCTCAATGTGCTAGCG |
| T8 (c.5407-5462) | TTTCCATCAATCAACTCCTCACCGTTGTTGGCTTGCACTGAAATTGCATTTATCGC |
| T9 (c. 5431-5455) | GCCAACAACGGTGAGGAGTTGATTG |
| T10 (c. 7201-7257) | CCACTTAATGTATGGCCCTGGGAGACCATTGAGAGCATC AAAACACAAACTCGTGTC |
| T11 (c. 7223-7248) | CTCTCAATGGTCTCCCAGGGCCATAC |
| T12 (c. 8883-8895) | CTCACTATAGGGAATATTAAGCTTGGTACCAATTGGACCACGCGTTTTTTTTTTTTTTTTTTTTTTTTTT |
| T13 (c. 1706^1707) | ACCGGGAATGGAAATGTGTGTAAATTGGATGTAAGTTTCTGCTTCTACCTTTG |
| T14 (c. 1706^1707) | ATTTGAATAGCTACTACTACACACTCTCAAGCTGCATATCAACAAATTTTGGTC |
| T15 (c. 3493^3494) | GATGCTGTTGGAATTTGTAGTTCTTACTTGGTAAGTATGCACTTAAAGAGTATG |
| T16 (c. 3493^3494) | CACTCGTCTCCATTCGCTCATATTTGTGGCCTGCATAATTTCAAAGATTGAACC |
| T17 (c. 5628^5629) | GCATTTTGAGTAAACCGGCGTTTAGGTAAGTGGATTCTCTTATAAATTTTGC*C* |
| T18 (c. 5628^5629) | CAGGTTCATTATATTTCAGCAAACCCTTCCTGCATTTGCCAAACACTTATCACTTACTTGCCAACTAGAT |
| T19 | AGTCAGATCTACCATGGTGGACTCCTCTTAAAGCTTGCATGCCTGCAGGT |
| T20 | TTCTTATGTTTTCTTATGTCATAGTTTAAATTTCTCTCCAAATGAAATGAACT |


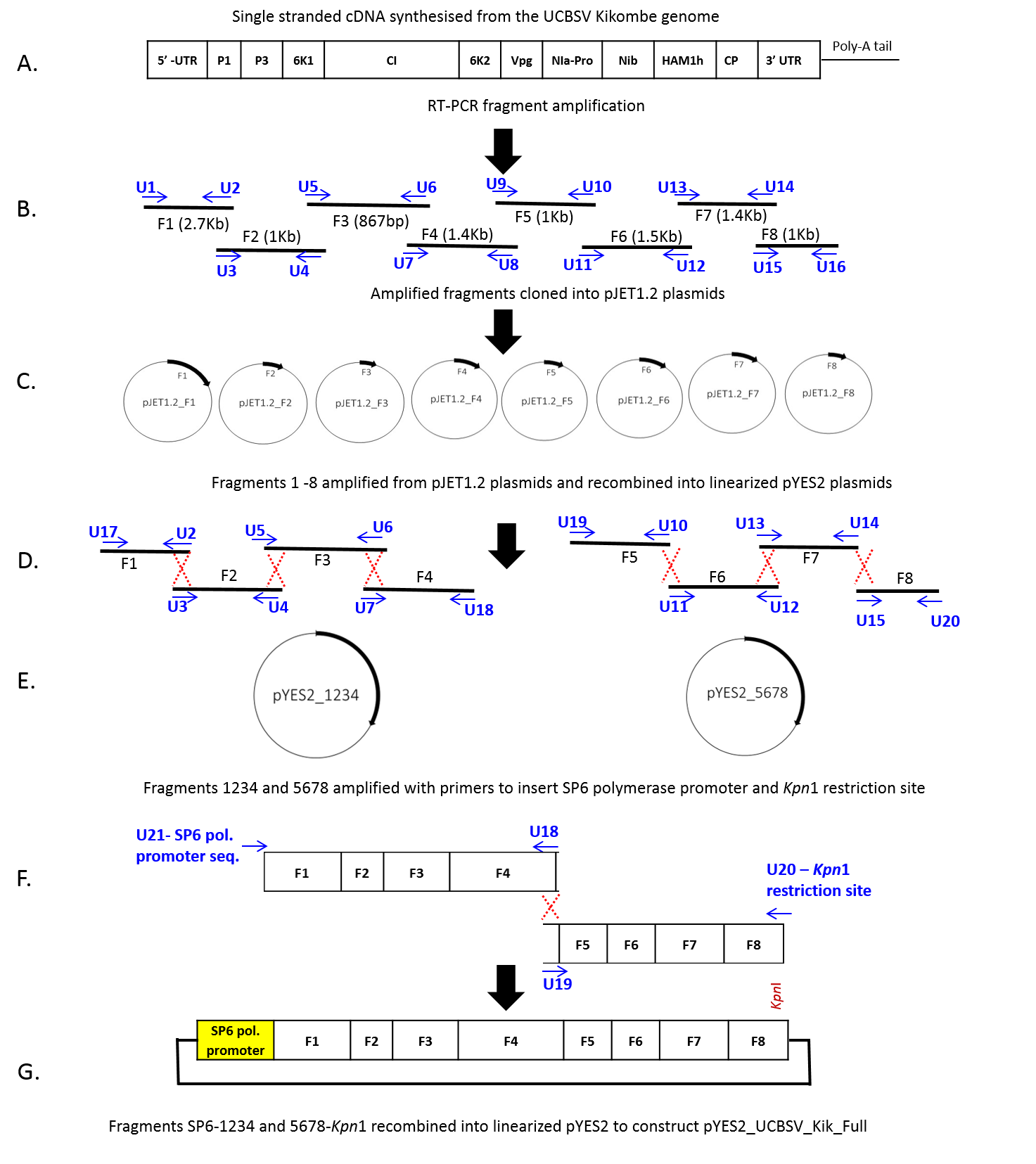


**Figure S1.** Cloning strategy for the construction of UCBSV ‘Kikombe’ IC

A. Single stranded cDNA was synthesized from cassava material infected with UCBSV ‘Kikombe’ using the oligo(dT)_18_ primer.

B. The cDNA was used as template for RT-PCR to amplify eight overlapping fragments with primers U1 – U16 (Table S2).

C. Each RT-PCR fragment was cloned into pJET1.2 and sequenced.

D. The overlapping fragments were amplified by PCR from pJET1.2 plasmids using the same primers, except fragment 1 was amplified with the U17 in the forward direction and fragment 8 was amplified with U20 in the reverse direction to introduce 5′ and 3′ homologous pYES2 sequence to fragments 1 and 8 respectively.

E. Amplified fragments were recombined into linearized pYES2 through homologous yeast recombination to form pYES2_Kik_1234 and pYES2_Kik_5678.

F. The two halves of the UCBSV Kikombe genome were amplified by PCR using plasmids pYES2Kik_123 and pYES2Kik_5678 as templates. The SP6 polymerase promoter sequence was added to the 5′ of the viral genome using the forward primer U21 to amplify fragment 1234. The Kpn1 restriction site sequence was added to the 3′ of the genome using the reverse primer (U22) to amplify fragment 5678.

G. The two halves of the genome were recombined through homologous yeast recombination to form pYES2_UCBSV_Kik_Full. Once digested with Kpn1, the full-length viral sequence served as template for SP6 polymerase *in-vitro* transcription


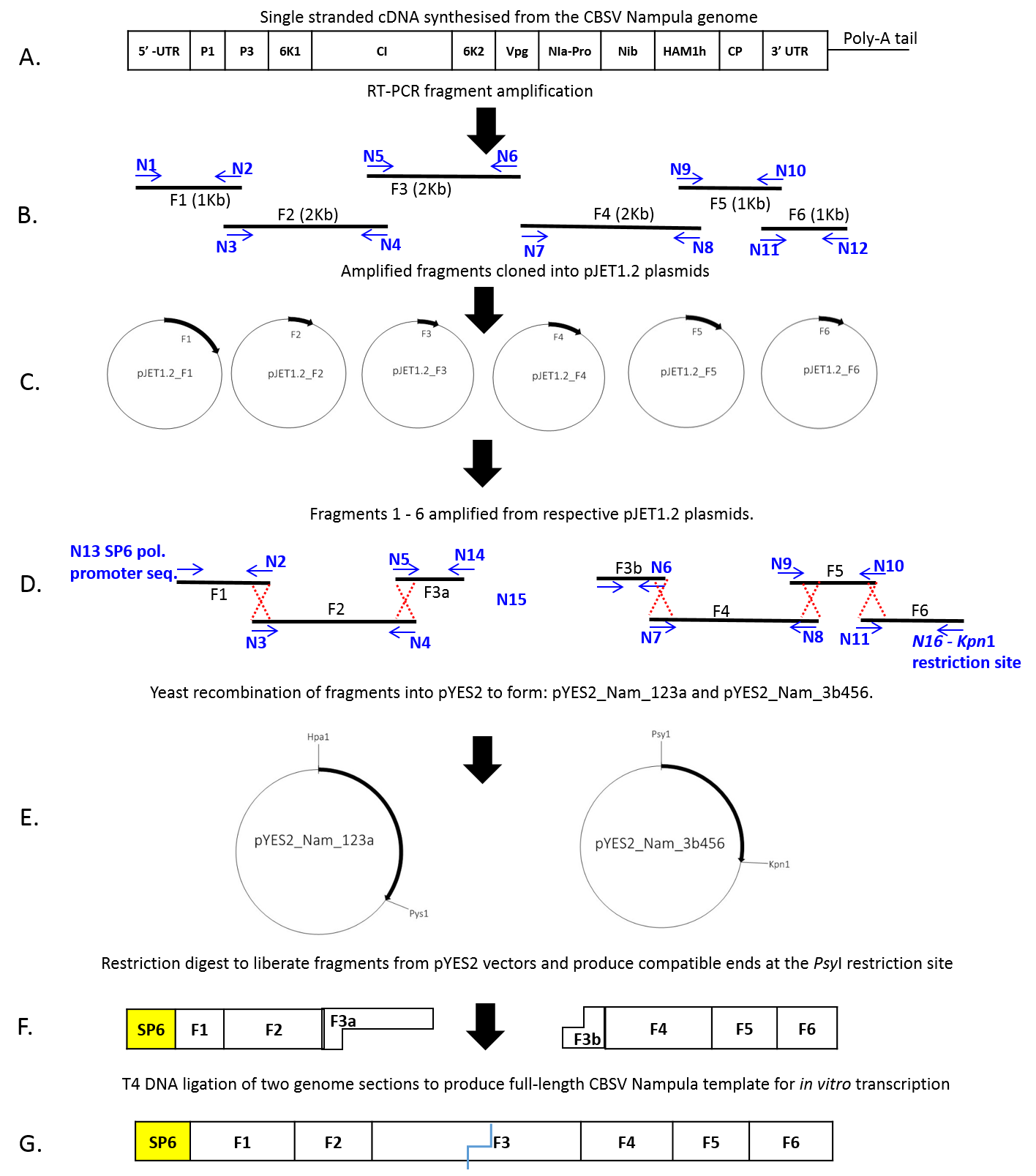


**Figure S2.** Ligation of two halves of the CBSV ‘Nampula’ genome, followed by *in vitro* transcription

A. Single stranded cDNA was synthesized from freeze-dried *N. benthamiana* material infected with CBSV ‘Nampula’ using oligo(dT)_18_ primer.

B. The cDNA was used as template for RT-PCR to amplify six overlapping fragments using primers N1– N12 (Table S2).

C. Each RT-PCR fragment was cloned into pJET1.2 and sequenced.

D. Sequence instability was detected in fragment 3 and so the cloning design split the viral genome into two halves, fragment 123a and 3b456, to stabilise plasmid propagation in *E. coli*.

E. The overlapping fragments were amplified by PCR from pJET1.2 plasmids using primers listed. Fragment one was amplified with the N13 forward primer, which contains SP6 polymerase promoter sequence. Fragment 6 was amplified with the N16 reverse primer, which contains Kpn1 restriction site sequence and pYES2 homologous sequence.

F. The RT-PCR fragments were recombined into linearized pYES2 plasmids through homologous yeast recombination to form pYES2_Nam_123a and pYES2_Nam_3b456.

G. The pYES2Nam_123a plasmid was digested with Hpa1 and Psy1 restriction enzymes and pYES2_Nam_3b456 was digested with Psy1 and Kpn1 restriction enzymes to liberate the two fragments: SP6-123a and 3b456 from pYES2 and produce compatible ends at the PsyI restriction sites.

H. The two fragments were ligated *in vitro* using T4 DNA ligase to produce full-length sequence, which served as template for SP6 polymerase in vitro transcription.

**
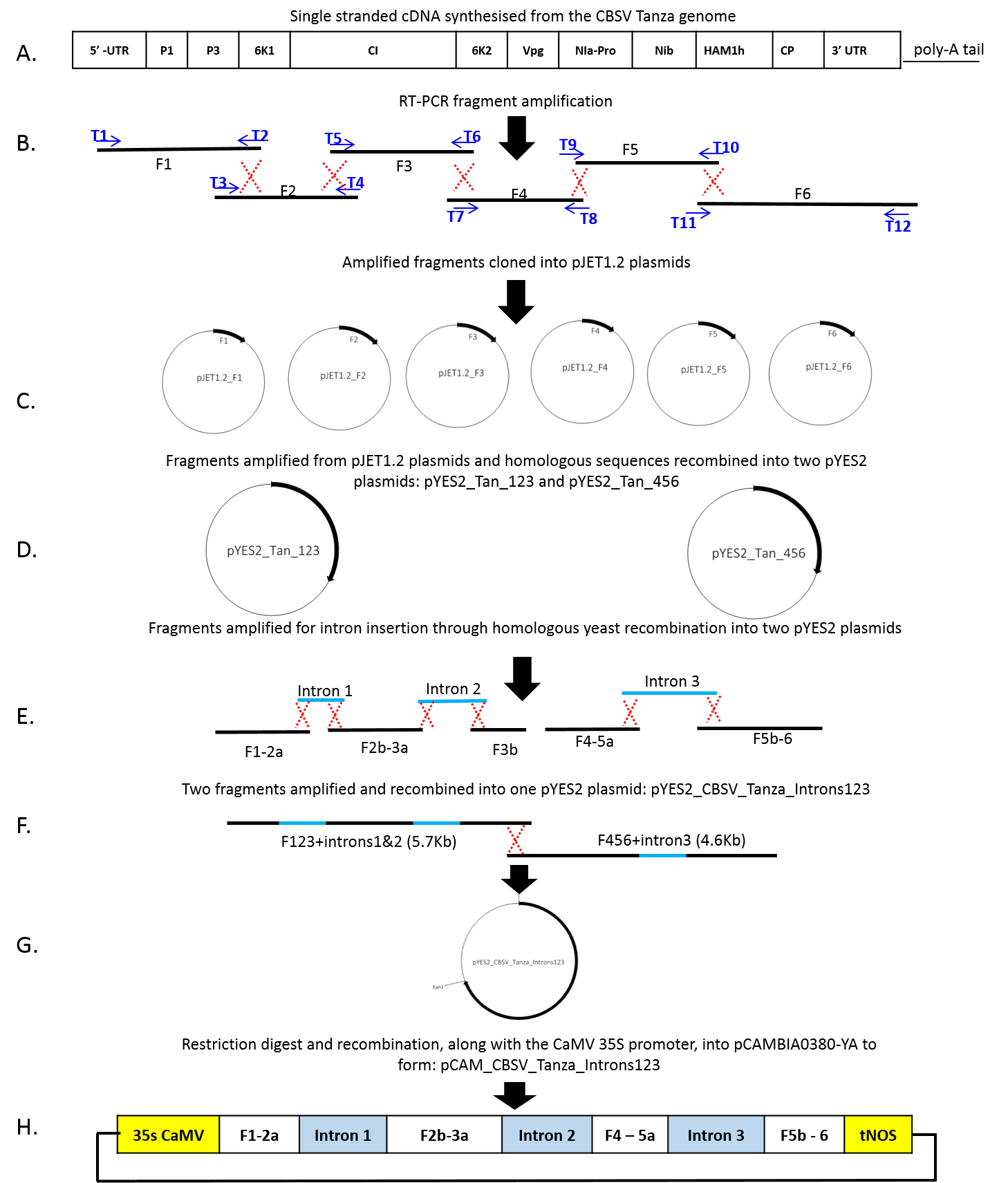
**

**Figure S3.** Intron insertion and construction of an *Agrobacterium* expression cassette for the CBSV ‘Tanza’ isolate.

A. Single stranded cDNA was synthesized from *N. benthamiana* material infected with CBSV Tanza using oligo(dT)_18_ primer.

B. The cDNA served as template for RT-PCR to produce six overlapping fragments with primers T1–T12 (Table S2).

C. Each RT-PCR fragment was cloned into pJET1.2 and sequenced.

D. To overcome sequence instability issues, F1, 2 and 3 and 4, 5, 6 were amplified by PCR and recombined into two separate pYES2 plasmids to form pYES2_Tan_123 and pYES2_Tan_456.

E. Overlapping PCR fragments were then amplified to enable the recombination of intron sequences into the unstable regions of pYES2_Tan_123 and pYES2_Tan_456. Fragments containing intron sequences 1 (186bp) and 2 (221bp) were inserted into the P1 and CI regions respectively of pYES2_Tan_123 and intron 3 (507bp) was inserted into the NIb region of pYES2_Tan_456 through yeast recombination to form pYES2_Tan_123_Introns12 and pYES2_Tan_456_Intron3.

F. The two plasmids pYES2_Tan_123_Introns12 and pYES2Tanza_456_Intron3 were used to amplify PCR fragments: F1-2a-intron1-2b-3a-intron2-3b and F4-5a-intron3-5b.

G. The two fragments were recombined into the linearized pYES2 vector to form pYES2_CBSV_Tanza_Introns123.

H. The intron containing sequence was liberated from pYES2 by restriction digest and recombined, along with the CaMV 35S promoter sequence, into linearized pCAMBIA0380.
